# Classic and Modern Meridian Studies: A Review of Low Hydraulic Resistance Channels along Meridians and Their Relevance for Therapeutic Effects in Traditional Chinese Medicine

**DOI:** 10.1155/2015/410979

**Published:** 2015-03-02

**Authors:** Wei-Bo Zhang, Guang-Jun Wang, Kjell Fuxe

**Affiliations:** ^1^Institute of Acupuncture & Moxibustion, China Academy of Chinese Medical Sciences, Beijing 100700, China; ^2^Department of Neuroscience, Karolinska Institute, 17177 Stockholm, Sweden

## Abstract

Meridian theory is one of the core components of the theory of traditional Chinese medicine (TCM). It gives an integral explanation for how human life works, how a disease forms, and how a therapy acts to treat a disease. If we do not understand the meridians, it is hard to understand the TCM. People in China and abroad had been working hard for 50 years, trying to understand the meridians; then 15 years ago a breakthrough idea appeared when we realized that they are low resistance fluid channels where various chemical and physical transports take place. The channel is called low hydraulic resistance channel (LHRC) and the chemical transport is named volume transmission (VT). This review aims to give a full understanding of the essence of meridian and its works on the therapies of TCM.

## 1. A Brief Review of Classic Meridian Theory and Modern Meridian Study

### 1.1. The Description of the Meridian Theory in TCM

According to TCM, there is a hierarchy network called meridian system existing in our body. It includes three grading levers: meridians, collaterals, and subcollaterals. The term of meridian or meridians is just a symbol to represent the whole system internationally. The main part of the system is the different sizes of channels, meridian channels, collateral channels, and subcollateral channels. There are 14 meridian channels (main channels) distributed longitudinally on human body while the collaterals and subcollaterals are smaller branched channels extending from the meridian channels which have much more amount than meridian channels.

The other important concept related to the channel system is Qi-Blood. As the essential substance and energy of the body, it flows in the channels and can spread throughout all organs and tissues and cannot be absent anywhere. Actually, Qi and Blood are two separated substances and flow in different channels: Blood-channel and Qi-channel. According to Yellow Emperor's cannon, Blood flows in vessel which is coincident with the blood vessel we know well while the large blood vessels are not totally distributed along the meridian lines according to the anatomy we have known. Qi, mainly Wei-Qi, flows in the interspaces of muscles or the interstices between different tissues such as bones, muscles, and vessels which are mostly distributed longitudinally in our body. An expected question is that if the Qi-channel or interstices match the 14 meridian lines. Xie et al. in 2009 published the anatomical study of interspaces of muscles that all 14 meridian lines are located in the connective tissue among the interspaces of the muscles [1]. Langevin and Yandow studied the relationship between meridians and connective tissue using an ultrasound scanner and got a similar result [2]. Such longitudinal interstices following the meridians were named “meridian interstices” by Zhang in 2000 [3] which are thought to be an important concept in TCM.

**Figure 1:**
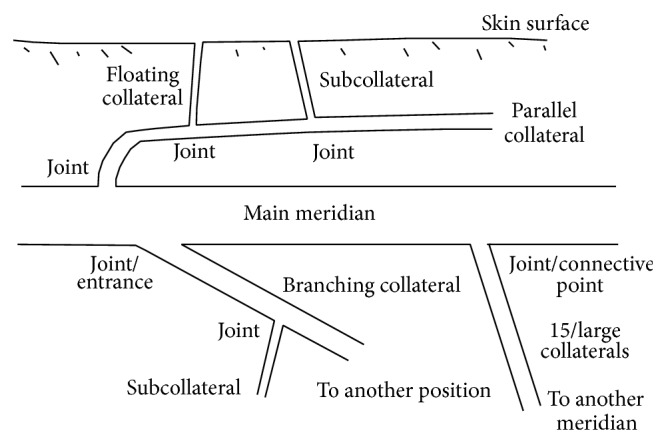
The illustration of meridian system. Joint is the position of acupoint which is also the entrance of Qi-Blood in or out of the channels. Floating collateral is one of subcollaterals toward skin surface. There are different kinds of collaterals, 15/large collaterals, branching collateral, and parallel collateral divergent from main meridians [5].

Another concept related to the meridian is acupoint. According to the first chapter of Lingshu in Yellow Emperor's cannon, acupoint, named Jie (Joint) is not any entitative tissue like skin, muscle, tendon, or bone but the entrances through which Qi-Blood come in or go out of the meridian channels [4]. For 365 acupoints on 14 meridians, there are 365 subcollaterals linked to the 14 meridian channels. The joints between the subcollaterals and meridians are just the location of acupoints (Figure 1).

Acupuncture is the therapy invented two thousand years ago which specially focus on regulating the flow of Qi-Blood in meridian-collateral channels. Acupuncture in narrow sense is the manipulation using filiform mental needle while moxibustion, cupping, massage, knife-needle, and so forth all belong to the acupuncture therapy because of the similar principle of regulating Qi-Blood. So meridian, Qi-Blood, acupoint, and acupuncture constitute four basic concepts in TCM which are very important to understand the principle of acupuncture.

There are also additional structures including meridian-sinew and skin meridian which are not channel-like structure. Meridian-sinews consist of a series of muscles and tendons which are linked to each other to form 12 meridian-sinews around the meridian channels. Skin meridians are the superficial part of meridians. There are 12 skin meridians where many subcollaterals coming from meridian channel distribute. Meridian-sinew and skin meridian are not responsible for the flow of Qi-Blood but can influence and support the flow. Skeleton system in our body is also related to the meridians. In the chapter of GuDu (Chapter 14 of Lingshu) in Yellow Emperor's cannon, long bones and joints were used to determine the meridians and could be regarded as a supporting structure of meridians as well.

The numbers of meridians, 11, 12, or 14, are just coincident with the number of viscera or match the numbers in Chinese numerology. It should be emphasized that meridian, collateral, and subcollateral are not anatomic concepts but grading concepts which may change to the anatomic concepts only when they combine themselves with the entitative structures, that is, vessel, interstice, muscle, skin, and so forth. The numbers of such anatomic structures are actually not limited to the number of meridians. The well-organized concepts of meridian system are summarized in Table 1 [5].

### 1.2. The Modern Research of the Meridian System before the 1990s

There were only scattered studies on meridian system before the 1960s when Western medicine was introduced into China. Scholars at that time naturally compared the anatomy in Western medicine and anatomy in TCM. Some people found the meridian system to be similar to the blood vessel system and deduced that meridians are arterial vessels and collaterals are venous vessels [6]. Another view regarded the meridian system as a mistranslation of the nervous system [7]. The real national organized study of meridian originated from the 1960s when Dr. Kim Bonhang from North Korea announced the discovery of the meridian as an anatomical structure and named it “BonHang tube” or “BonHang body” in 1963 [8]. Exactly in 1964, 50 years ago, a large group of scientists in China were selected to repeat the results from North Korea but failed to do it [9]. This group together with the other group who studied acupuncture anesthesia founded the institute where the authors stayed to continue the study of acupuncture and meridian mainly using morphological and physiological methods. Later on, a phenomenon called propagated sensation along meridians (PSM) was found in acupuncture clinic and a wide investigation was carried out. In a group of 1000 cases, 1.3% patients were found to have obvious PSM, 1.8% had relatively obvious PSM, 15.2% had few PSM, and others (81.7%) had no PSM [10]. PSM has characteristics of low speed, going toward afflicted sites, and being blocked by physical pressure which is difficult to be explained by known neural and blood transmission. It implied a role of meridian channels.

After the existence of PSM was confirmed, a program for tackling key problems was carried out. A deeper study of meridian was carried out and several physical features of the meridian line were found such as the low electric impedance, high vibration and conductance of sound, and migration of isotopes along meridians. All these studies implied the existence of an unknown system different from any known anatomic structure. Afterwards, the study focused on the explanation of meridian phenomena in the following two “five national key projects” conducted by the national ministry of science and technology and several hypotheses were put forward including mainly three aspects represented by neuron, body fluid, and electromagnetic field [11]. But none of the aspects separately gives an integrative explanation of meridian phenomena and complies well the classic meridian theory before 1990s. So an exploration should be done to build a new model and integrate all the studies which is just the aim of this review.

## 2. A Discovery of Low Hydraulic Resistance Channel along Meridians

### 2.1. A Hydromechanical Model of Interstitial Fluid Flow along Meridians

During the investigation of PSM, some people described that a flow-like sensation moves along meridians and this feeling can be blocked and become thicker when mechanically pressing the route of the meridian. In acupuncture clinic, when a doctor wants the needle feeling moves faster in one direction, he can simply press the opposite route of the meridian. Such phenomena give clues on a flow of fluid in meridian channels. Migration of isotope along meridians (MIM) on the other hand more strongly demonstrates the existence a flow in meridians. As no vessel-like structure was found along the meridians, a nonvessel flow of interstitial fluid (IF) was introduced by Zhang based on the Darcy's law and conservation equation.

Darcy's law (1856) describes the average velocity of a liquid as

(1) $$\begin{array}{c} \overset{-}{V}=-\frac{k}{\mu }\nabla P, \end{array}$$

where “*k*” represents the permeability of tissue which is positively related to the number of pores and the connectivity in tissue or negatively related to the tortuosity of the pores in tissue. “*μ*” is the dynamic viscosity coefficient of the liquid. “∇*P*” is the pressure gradient in the fluid.

Conservation equation requires three velocity components (*u*, *v*, *w*) to be

(2) $$\begin{array}{c} \frac{\partial u}{\partial x}+\frac{\partial v}{\partial y}+\frac{\partial w}{\partial z}=0, \end{array}$$

which means the liquid cannot be compressed; namely, the inflow should be equal to the outflow in any space.

**Figure 2:**
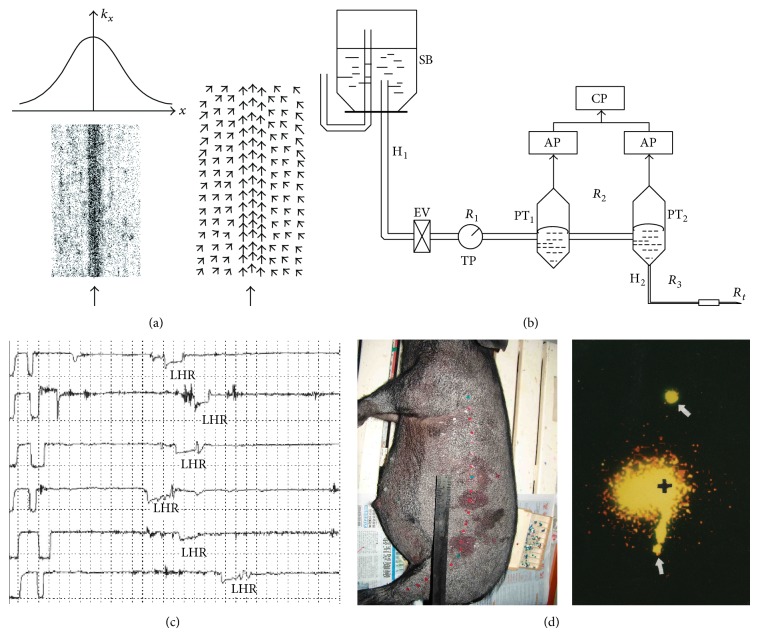
Hydromechanical model of meridian and the experimental verifications. (a) Higher permeable (*k*_*x*_) tissue (low HR) along meridian (↑) and flow pattern of IF of the meridian. (b) The measuring system of HR with two pressure transducers. (c) The HR curve and low HR points (LHR) on stomach meridian in a minipig. (d) The sites of isotope injection (middle green point in left figure and black cross in right figure) and the migration of the isotope along LHRC of stomach meridian (marked by two green points in left figure and white arrow in right figure) [20].

The model assumed that there is better permeability, that is, lower hydraulic resistance (HR) along the meridian; the IF will move toward the meridian from both sides and flow along the meridian because of the incompressible feature [12] (Figure 2(a)). This model can be generalized to any nonvessel channels including meridian, collateral, and subcollateral channels.

### 2.2. Verifying the Low Hydraulic Resistance along Meridians

The critical verification of the hypothesis is to measure the hydraulic resistance and to see if there is lower resistance along meridians. Levick is the rare scholar who studied hydraulic resistance in biological tissue in all his life. He developed a method to measure the resistance by dropping saline into a tissue through a reservoir and counting the drop per minute [13]. A method which is quite similar to Levick's was used to measure the resistance on and outside the meridians. The result supported that the tissue along meridian is a more permeable tissue [14]. But this method causes an accumulation of liquid in tissue which may disturb the result. Guyton et al. from America have measured the hydraulic conductance using a pressure differential technique which can overcome the problem of accumulation of liquid in the 1960s [15]. A modified Guyton technique was used to measure hydraulic conductance in 15 rats and 4 minipigs. The results showed that the conductance (an inverse of resistance) along the meridian line, determined by low electric impedance, is significantly higher versus the conductance along the nonmeridian area [16].

### 2.3. Discovery of the Low Hydraulic Resistance Channels along Meridians

As the measurement of hydraulic conductance should be taken by two parallel needles with difficulties to locate the site of the lowest resistance, a new method was built up to measure the resistance using a single WIN needle connected to two pressure transducers (Figure 2(b)). A low hydraulic resistance point (LHRP, a concave episode in the HR curve) could be found when moving the needle crossing the low impedance meridian line and a series of LHRPs could be observed (Figure 2(c)) which may reveal a low hydraulic resistance channel (LHRC) along meridians [17]. It led to the question if LHRPs consist of a continuous channel in the subcutaneous tissue. As it is impossible to measure the LHRP side by side with an infinitely small distance, a new technique was developed to detect the connectivity between the LHRPs. A fluid pressure wave is produced by injecting a small amount of saline into one LHRP and pressing the site with a 200 g weight. Three pressure transducers were set to measure the pressure wave on two LHRPs and a point beside LHRP with higher resistance. The experiment has been done on conceptual vessel meridian of six rats and the result showed a significantly higher pressure transmission along the meridian [18]. The same measurement was carried out on stomach meridian in another group of five rats and similar result was obtained [19].

### 2.4. Isotopic and Morphologic Study of LHRC

The experiments of pressure transmission proved a better connectivity, likely continuous between the LPCPs to form a channel-like structure along the meridians. But it is hard to confirm the channel by indirect measurements of resistance or pressure wave. Morphological and image presentation is always the direct evidence for a structure. As the channel cannot be separated from the tissue, it can only be demonstrated by colored materials which can move in the channel. Isotope is a material which can emit *γ* ray all the time. The *γ* ray can be collected by a *γ* camera, which gives the position of the isotope. The isotope ^99m^TcO_4_ was injected into one LHRP on six minipigs and a track of isotope can be seen through the camera (Figure 2(d)) [20]. The similar observation has also been made in the human body in a large number of subjects and all the fourteen meridians have been presented by the technique [21]. Alcian blue (AB) is a dye which is easy to combine with hyaluronic acid, the main substance in the interstitium. We injected small amounts of AB slowly into LHRP of pigs and recovered the skin 1 hour after the injection. Linear migration of AB could be found in the minipigs, 1~6 cm long and several micrometers in width [20].

### 2.5. The Study of IF Channel with MRI Technique

As the resolution of *γ* camera is very low and can only be seen in two dimensions, MRI was used recently by doctor Li et al. to visualize regional hypodermal migration along channels of IF in human beings. The author injected minimal amount of tracer, gadolinium diethylenetriamine pentaacetic acid (Gd-DTPA), into six acupoints on six Yin meridians of hand and foot and six regional migration tracks could be observed originating from injected points by MRI. Acupuncture needling was used to check the channel which showed a different response from blood vessels and lymph vessels, suggesting that an open channel exists along the meridians [22].

## 3. The Physical Features of LHRC

### 3.1. IF Pressure of LHRC

According to the hydromechanical model of IF, the flow goes on under the condition of pressure gradient. IF pressure (IFP) is a physiological parameter which is difficult to be measured as the fluid scatter in the tissue. Several techniques were developed such as capsule technique, needle technique, wick technique, wick-in-needle (WIN) technique, and micropipette technique [23]. Although the last is the best, it can only be used on very superficial tissue. So WIN technique was used in measuring the subcutaneous IFP. On anesthetized minipigs, low hydraulic resistance points (LHRP) and non-LHRP were measured by a scanning hydraulic resistance measuring device. The IFP was then measured by wick-in-needle method on these two regions. The stomach meridian, kidney meridian, and conceptual vessel meridian were measured where the IFP was significantly lower than in non-LHRP region along the three meridians (*P* < 0.05); the differences were 1.06, 0.70, and 3.69 (mmHg), respectively, with the sum of 1.44 mmHg and 1.44~2.88 mmHg/cm pressure gradient. The experiment proved that there is a lower IFP in LHRC and a pressure gradient toward LHRC along meridians [24] which gave a dynamic source of convergent flow of IF toward the meridians.

Zhang et al. studied the distribution of IFP with micropipette technique on hind-limb skin of rats and found a significantly lower IFP on the concave area versus the convex area. The concave surface is the characteristic of most acupoints [25].

### 3.2. Impedance along LHRC

Low electric impedance (LEI) is an important feature observed on human meridians by many researchers and has been systematically reviewed by Ahn et al. recently [26]. Although different methods were used such as 3~12 DC, single power alternative current, narrow pulse, and constant current, the results are almost the same and show the low electric impedance feature along meridians or at acupoints [27, 28]. The mechanism of LEI was studied by Ahn et al. using ultrasound detector and attributed LEI to connective tissue planes [29]. The other researcher Yang in China explained the LEI by rich IF within loose connective tissue [30]. As connective tissue and IF are correlated, both scholars got similar answers. This is also in agreement with results from other biophysical scientists who estimate the volume of IF by measuring the impedance with four electrodes and constant current called bioelectric impedance analysis (BIA) technique [31].

To further verify the relationship between LEI and IF, we made a fluid model by embedding a bunch of fine nylon fibers into a melted gel and carefully withdrawing them after the gel became hard. We then injected slowly a small amount of saline into the porous medium channel. A fluid channel among the gel plane was successfully made, simulating the real situation of meridian channel on human body.

Firstly, the low hydraulic resistance was confirmed using the instrument mentioned above (Section 2.3, [17]) on the model. The impedance on the channel was measured by a four-electrode impedance instrument with 5 kHz constant current which showed a significantly lower impedance on the fluid channel than adjacent area (*P* < 0.05) [32]. The result supports the speculation by Zhang et al. that a rich IF in meridian channels causes the low impedance. Also the low impedance point located with single power alternative current showed lower impedance measured by four-electrode method [28].

### 3.3. Transmission of Sound Wave along LHRC

During the 1990s, some physical features were found along human meridians, one of which is the good transmission of sound wave along meridians. We examined the feature on the fluid channel in the gel plane mentioned above. A vibration generator was put on one set of the channel to produce 50 Hz sound wave. The signal was detected by a crystal pickup on the other set of the channel. The result showed a better transmission of the sound wave along the channel than that in nonchannel (*P* < 0.05).

The results from the above two experiments illustrate that LHRC has features similar to the features on human meridians and may be the essential part of meridians [32].

## 4. The Biological Functions of LHRC

Meridian and collaterals have the function of running Qi-Blood, balancing Yin-Yang, nourishing tissue, smoothing joints, and communicating between organs and limbs. How do you understand these functions based on the modern scientific concepts and knowledge?

### 4.1. The Transportation of Hot Water along LHRC Observed by Thermal Infrared Imager

**Figure 3:**
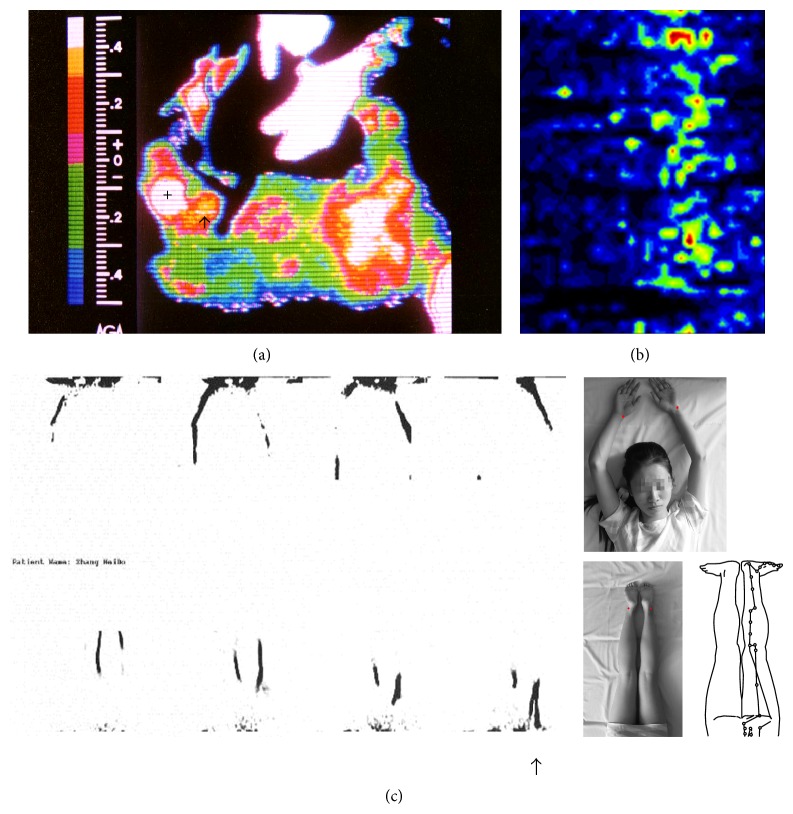
The functions of LHRC along meridians. (a) A higher thermal line along stomach meridian was shown (↑) by thermal infrared imager after injecting hot water into Zusanli (+). (b) A zone of high blood perfusion appeared 30 min after injecting histamine into LHRC. (c) The migration of  ^18^F-FDG along lung meridian and heart meridian (upper four figures) and along bladder meridian (low four figures). ↑ indicates a branch of  ^8^F-FDG along bladder meridian separated centripetally at the middle of thigh. The figures on the right show the postures of the subjects. The red points are the injecting points. The rightmost line chart shows the branch of bladder meridian on thigh.

To see if the channel can really transport water, 1 mL of 50~60 hot water was injected into a low HR point near Zusanli (ST36) along the stomach meridian and the thermal change on the skin surface was observed by a thermal infrared imager (AGA-782, made in Sweden) in four LHRCs on two minipigs. A short high thermal line about 3 cm long could be found along the meridian in one case (Figure 3(a)). As the heat of water diffused rapidly, the hot line is not very long but can somehow illustrate the ability of transporting water in LHRC.

### 4.2. The Transportation of Histamine in LHRC

If meridian is a kind of channel, Qi may be represented by active chemicals which can cause biological effects. Histamine was chosen to be studied as it is released from mast cells and has important functions related to the meridian and acupuncture [3]. We examine the transportation of histamine in LHRC on four anesthetized minipigs by injecting 0.1 mL histamine of 1% into one low hydraulic resistance point and observe the blood perfusion (BP) on the skin where the meridian passes across. The result showed a significant increase of BP in meridian area compared to the adjacent nonmeridian area and the situation when injecting 0.1 mL saline [33]. A zone of high blood perfusion along the meridian could be seen clearly 30 min after the injection (Figure 3(b)).

### 4.3. The Transportation of Glucose along Meridians

Glucose is the main molecule to bring life and growth in our body. To observe if meridian has the ability of transporting glucose, positron emission tomography (PET) was used on four healthy subjects. ^18^F-FDG of 50 *μ*L, 0.1~0.5 mCi, was injected subcutaneously in Kunlun (BL60) of bladder meridian on both sides simultaneously in two subjects. In another two subjects, Shenmen (HT7) of heart meridian on left arm and Taiyuan (LU9) of lung meridian on right arm were injected simultaneously. Isotopic tracks were found along the meridians in all the subjects. In one subject, a centripetal branch of bladder meridian was even observed (Figure 3(c)) which cannot be explained by any structure except bladder meridian. Similar studies were carried out by Zhu et al. [34] on other human subjects and isotopic tracks could be found in most cases.

### 4.4. The Transportation of Furosemide along LHRC to Get a Better Effect

Point-injection therapy has been widely used in acupuncture clinic which has a good curative effect. To see if an injected medicine exerts its effect through meridian channel, an acute edema model was established in minipigs through rapid vein injection of 2000 mL saline. Furosemide was injected with full quantity in vein and Zusanli (ST36). Low HR point on Shuifen (CV9) was injected with half-furosemide or half-saline. Urine was measured to study the effect of furosemide. The result showed that there were similar higher effects on vein group and Shuifen of furosemide group at 15–30 min after giving the medicine while the other two groups have much lower effects at the time which implied that a medicine has better effect through the transportation along LHRC, giving an explanation of point-injection therapy [35].

## 5. The Pathological Study of LHRC

A fundamental difference on pathology between Chinese medicine and Western medicine is the mechanism of forming a disease. Usually a bacterium or virus is the reason to cause a disease in Western medicine while a channel stasis and then Qi-Blood discordance are the cause of a disease in TCM. Such a big difference in pathology and etiology between the east and the west directs a doctor's treatment in quite a different way, killing the bacteria versus dredging the channel. We wonder if the channel stasis is the real reason for a disease.

### 5.1. Establishing the Model of Meridian Stasis

Blocking technique is an important method to study the function of a structure in biology. Polyacrylamide hydrogel which is quite similar to the component of hyaluronic acid in interstitium is used as the blocking substance of the channel because hyaluronic acid has very high hydraulic resistance according to the study of Coleman et al. [36] McDonald and Levick [37]. To establish the model of meridian stasis, the hydrogel was injected into LHRC. The transmission of IFP wave was measured by the method described in Section 2.3. The results showed that there was a significant decrease (*P* < 0.01) of IFP wave when injecting more than 0.5 mL hydrogel into the channel while no significant changes were found when injecting saline and injecting the hydrogel outside the channel. The experiment proved that LHRC can be blocked by injecting certain amount of polyacrylamide hydrogel and the pathological model of obstructed channel was preliminarily established [38].

### 5.2. The Change of Pain Threshold after Blocking the Channel

Pain is the common symptom in a disease. According to the theory of TCM, pain appears whenever the channel becomes stasis. To see if blocking a channel represented by LHRC can really cause pain, an injection of gel into the channel was carried out on 12 anaesthetized minipigs. Tail-flick threshold and ear-flick threshold were measured immediately, 2 days and 4 days after the injection using a thermal radiation dolorimeter and relative flick threshold (RFT) was obtained. The result showed that RFT was significantly decreased 2 days and 4 days after the injection while no significant changes were found when injecting saline or injecting gel outside the channel. The study supports a possible role of Qi-channel in forming a disease. The mechanism concerns an accumulation of algogenic substances in the channel because of its blockage which has been emphasized by Xuan, a famous doctor who has treated 60 thousands patients successfully under the instruction of the theory of aseptic inflammation as the cause of chronic pain [39, 40].

### 5.3. Verifying the Influence of Meridian to Corresponding Organs by Blocking LHRC

**Figure 4:**
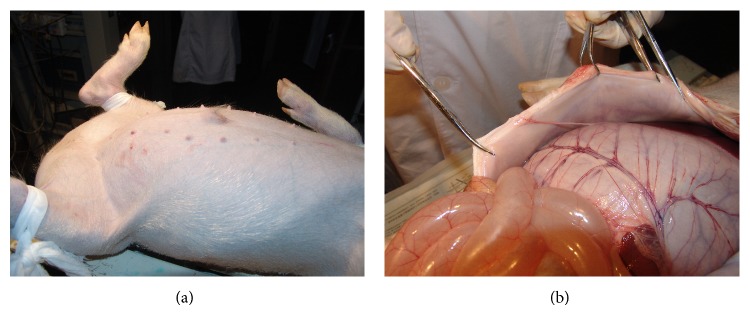
Pathological changes of gastrointestinal system after blocking the stomach meridian in a pig. (a) Before opening the abdomen which has been enlarged. (b) After opening the abdomen, distension of stomach and intestine could be found.

The interaction between meridians and organs (Zang-Fu) is one of the important aspects in meridian theory and is the guide of clinic acupuncture to treat the disease of internal organs. To see if a meridian can really influence the organ, we blocked the LHRC of stomach meridian in minipigs and feed them for 6–10 weeks to see what happens. The blocking model was built up on 8 minipigs by injecting gel into 6 to 8 points along LHRC and the same amount of saline was injected into the other 8 minipigs as a control group. The result gave changes of distention on stomach and/or intestine in all the blocked pigs (Figure 4) while no such changes were observed on the control pigs. The finding confirmed that the blockage of low hydraulic resistance channel along stomach meridian can influence the state of stomach and intestine in accordance with the classic meridian theory.

### 5.4. Muscle Contraction Increased the Hydraulic Resistance of LHRC

According to Yellow Emperor's Canon, meridian channel exists between the muscles. It could be speculated that the channel will become narrow when the muscles contract. To test the speculation, we stimulate the muscles with 1, 3, 5, 7, and 9 mA constant current and measured the HR of LHRC at different currents. Muscle contraction was scored by 1—no contraction, 2—rhythmic contraction, and 3—tonic contraction. The result on eight points in 4 minipigs showed that the HR significantly increased when the current rose to 7 mA and most muscles showed tonic contraction [41]. The result gave a mechanism for the influence of mind on the body. When a person gets stressed, the muscles are usually contracted continuously unconsciously and this will obstruct the Qi flowing in the channel and in turn weaken your health. So meditation can help you to be healthy by reducing the stress which has been proven worldwide [42].

## 6. Understanding Propagated Sensation along Meridians 

### 6.1. The Mechanism of Axon Reflex Relay on PSM

In Section 1.2, we have introduced the phenomenon called propagated sensation along meridians (PSM) which had been broadly investigated in the 1970s and many studies were centered around PSM afterward. Within many hypotheses, Zhang put forward the hypothesis of PSM as an axon reflex relay linked to the release of substance P (SP) and histamine [43]. The linked substances were increased by adding glutamate, ATP, and neuropeptide through the excellent work of Zhang et al. [44–46]. Great difference in this model from other neural aspects of PSM is that SP, histamine, and other signaling molecules have to diffuse and flow in the extracellular fluid (interstitial fluid) to the target cells which is called volume transmission [47], giving a chance to meridian channels to play a role in PSM.

### 6.2. The Mode of Volume Transmission between Neurons

VT is a widespread mode of intercellular communication which occurs through diffusion and flow in the extracellular fluid and cerebrospinal fluid. This concept was put forwarded by Fuxe and colleagues in 1986–1988 [48], based inter alia on early observations in 1970 [49, 50]. It was systematically reviewed as a theory in 2010 [51, 52]. During the process of VT, the signals move from source to target cells via energy gradients leading to diffusion and convection (flow). There exist a short distance and fast form, called extrasynaptic VT, and a slow long distance form which uses the paravascular and para-axonal pathways of the CNS in addition to CSF pathways [53, 54]. The paravascular pathways are also used for the clearance of interstitial solutes which was discovered by Iliff et al. recently using an in vivo two-photon imager technique [55]. The clearance system involves a para-arterial CSF pathway, an intracellular pathway over the astrocytes (aquaporin 4 channels), and a paravenous pathway.

### 6.3. The Mechanism of PSM Combined with Axon Reflex Relay, VT Theory, and LHRC

**Figure 5:**
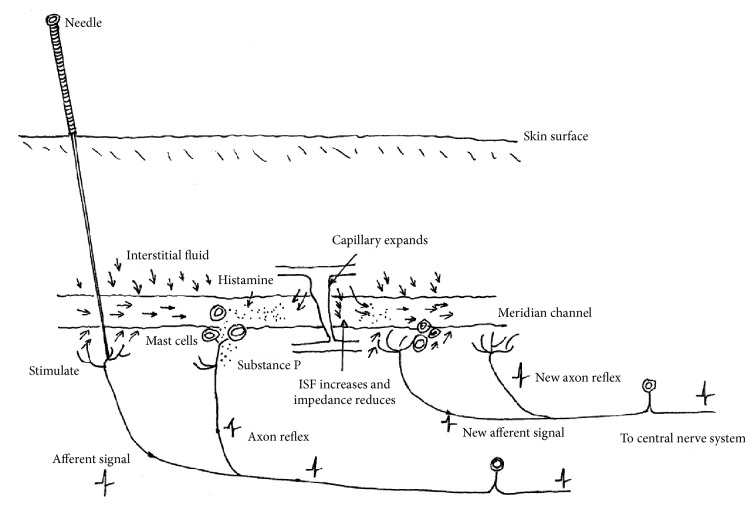
The mechanism of PSM involves axon reflex relay, volume transmission, and LHRC in peripheral tissue along meridians [56, 57].

To find a new field for VT theory, Fuxe began to be interested in acupuncture and has been the chief of selection committee of acupuncture and meridian study (AMS) award since 2008. He met Zhang in 2008 and knew his meridian study at that time and Zhang learnt Fuxe's VT theory simultaneously. Both scholars agreed that PSM could be well understood by VT in peripheral tissue along meridians. In 2012, Zhang invited Fuxe and Zhao to Beijing and discussed the mechanism of PSM intensely. They published a paper together to explain PSM by alternative axon reflex (wired transmission, WT) and VT in peripheral tissue in LHRC along meridians, sending simultaneously a continuous sensate signal to control the nervous system which can be felt like a propagated sensation along meridians [56] (Figure 5).

## 7. The Mechanism of Dredging Meridian Channel by Acupuncture and Other Therapies in TCM

### 7.1. Dredging Meridian Channel by Acupuncture

The real significance of a basic research in medicine is that it can explain logically the clinical effect and promote the effect by creating new therapy or improving the method. Acupuncture has been widely used in clinic in the world for 40 years while the explanation of acupuncture effect is poor. Neural mechanism of acupuncture is the one which is relatively well accepted but ignoring the existence of acupoints and meridians. According to the theory of TCM, acupuncture acts on dredging the channels which are blocked. To verify the ancient theory combined with the newly discovered LHRC, hydraulic resistance (HR) was measured in 13 low HR points and controlled high HR points on three anaesthetized minipigs before, during, and after the acupuncture. The result showed that at the later time, during acupuncture and after acupuncture, HR decreased significantly while no such change was found on control points. The mechanism of dredging the channel is similar to the mechanism of PSM as seen in Figure 5 in which the processes of increasing blood perfusion measured by laser Doppler perfusion imager in turn increase interstitial fluid volume measured by four-electrode impedance method that have been proven in Zhang's lab [57–59]. The study supports an effect of acupuncture on dredging meridian channels, giving a meaning of HR index as reflecting the degree of opening of the channels which is very important in the future meridian studies.

### 7.2. Understanding the Therapeutic Effects of Various Therapies in TCM

TCM is featured by rich natural therapies like acupuncture, moxibustion, cupping, scrapping, massage, and so forth. In the theory of TCM, a main principle for most therapies is the channel, Qi, and Blood. In most therapies, the principle of dredging channel and regulating Qi-Blood was recorded, leading to a balance of Yin and Yang. It is very difficult to explain the effects coming from the different therapies using a mechanism based on Western views rather than the hypothesis of IF channel of meridian.

#### 7.2.1. Moxibustion

**Figure 6:**
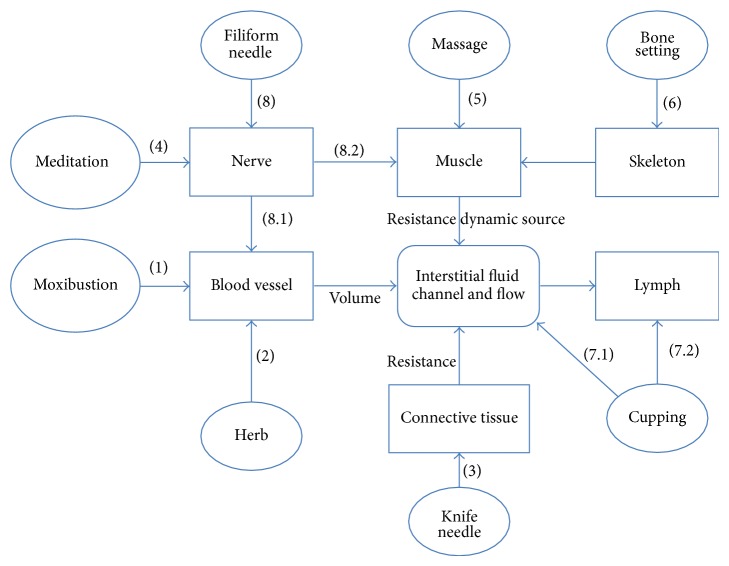
A sketch map to illustrate the mechanisms for therapies in TCM. The meaning of (∗) could be found in Section 7.2.

The conditions for IF flowing in a channel involve several factors. One is the volume of IF coming from blood circulation, mainly the capillary. So the increasing of blood perfusion or large opening of capillary will enhance the flow which is the main mechanism of moxibustion (Figure 6(1)). According to the recent experiment in our lab, moxibustion can greatly increase the blood perfusion on skin by more than 100% instantly after the moxibustion [60].

#### 7.2.2. Chinese Herb

In a recent study of X. Mu's group in Beijing Agriculture College, some Chinese herbal drugs used to activate blood circulation to dissipate blood stasis can enlarge the wave amplitude of blood perfusion [61] which can greatly benefit the flow of IF. But this might be just one of the mechanisms of Chinese herb in TCM (Figure 6(2)).

#### 7.2.3. Knife Needle

According to Darcy's law (1), the specific hydraulic conductivity (*k*) relates to the tortuosity of extracellular space which is the characteristic of tissue itself and can be shrunk by mechanical pressing. This characteristic let us understand the blockage of physical pressure in PSM. LHRC locates in loose connective tissue or fascial tissue between the muscles, muscle and skin, or muscle and bone. There is pathological adhesion between tissues, increasing the resistance of channel which is difficult to be separated by any method except a knife. A needle knife is specific for separating the adhesion and reducing the resistance of the channel (Figure 6(3)) and has been widely used in pain symptom, getting better effect than needle acupuncture in Wei's clinical study and better effect than pill treatment reported by Liu et al. in recent papers [62, 63].

#### 7.2.4. Psychological Therapy

According to the study of muscle contraction in Section 5.4, the resistance of a channel can be seriously influenced by the contraction of muscles particularly when the muscle contraction gets tonic [42] which often happens unconsciously in our daily life when we become nervous and depressed or keep a posture constantly, and so forth. Psychological therapies like meditation and breath exercise are getting popular in the west, which are called Qigong in China and Yoga in India. The main principle of such therapies is to let muscle relax through neural inhibition which can reduce the resistance of the channel between the muscles (Figure 6(4)).

#### 7.2.5. Massage and Bone Setting

Another factor to influence the flow of IF is pressure gradient, that is, ∇*P* in Darcy's law (1). If a muscle contracts rhythmically or a periodic external force acts on the channel, the flow of IF will be improved. Massage is not only popular in China but also in other Asian countries. The most characteristic of massage is the periodic external force which can provide the dynamic source to the movement of IF (Figure 6(5)). It can also relax muscles and release the fatigue through increasing IF flow which can remove the lactic acid within the muscles. Bone setting is one kind of massage which can regulate the positions between the bones and linked muscles that reduce the resistance of the channel between the bones and muscles (Figure 6(6)).

#### 7.2.6. Cupping Therapy

Cupping is the only therapy which can directly affect IF by producing more negative pressure in local tissue, causing a pressure difference both from surrounding area and from deeper tissue (Figure 6(7.1)). IF therefore flows and metabolic waste is driven from deeper tissue to superficial tissue which is more easy to be cleaned up by vascular system. It is extremely effective when moving a cup along a meridian which can produce a moving pressure gradient to drive IF flow in the channel. Cupping also increases the negative pressure in lymph vessel to enhance IF flowing into lymph vessel when putting the cup on large lymph node usually near the joints (Figure 6(7.2)).

#### 7.2.7. Filiform Needle

Acupuncture with thin metal needle is mostly used form in acupuncture clinic which is also called filiform needle therapy. The sensation of acupuncture, that is, Deqi, is important to get the effect, indicating sensory nerve involvement in the process. The mechanism is complicated as not only neural activation but also other factors like mast cell, connective tissue, capillary, and interstitial fluid are included. The mechanism through neuron, blood vessel, and IF mentioned in Section 7.1 is just one of the mechanisms of filiform needle (Figure 6(8.1)) which has been proven step by step. The other mechanism relates to the neural-muscle reflex of *α* motor neuron from muscle spindle receptors which may cause a rhythmic contraction to enhance the flow of IF (Figure 6(8.2)). It can also inhibit excessive contraction of muscle through negative feedback from the Golgi tendon organ that reduces the resistance of the channel.

A sketch map is drawn to illustrate the mechanisms for all these therapies in TCM (Figure 6).

### 7.3. Understanding the Therapeutic Effect of Acupuncture by Regulating the Milieu Interne through Interstitial Flow

Why increasing the flow of IF can get therapeutic effects? As everyone knows, all the cells live in a fluid environment with various solutes and physical conditions which is called milieu interne. The life and functional activity of a cell depend on mostly the homeostasis of the milieu interne. The recent developed epigenetics indicate the importance of milieu interne for gene expression. Milieu interne around cells is always polluted by metabolite coming from the cells while the circulation system works hard to keep the homeostasis of milieu interne by clearing up the metabolite and supplying nutrient. We usually think that blood flow and lymph flow play the role of keeping homeostasis of milieu interne while the interstitial flow is the actual leading role player to do the job. A metabolite can be regarded as a solute, the movement of which follows Fick's diffusion law:

(3) $$\begin{array}{c} N_{i}-X_{i}\sum N_{j}=-D_{i}C\nabla X_{i}. \end{array}$$

**Figure 7:**
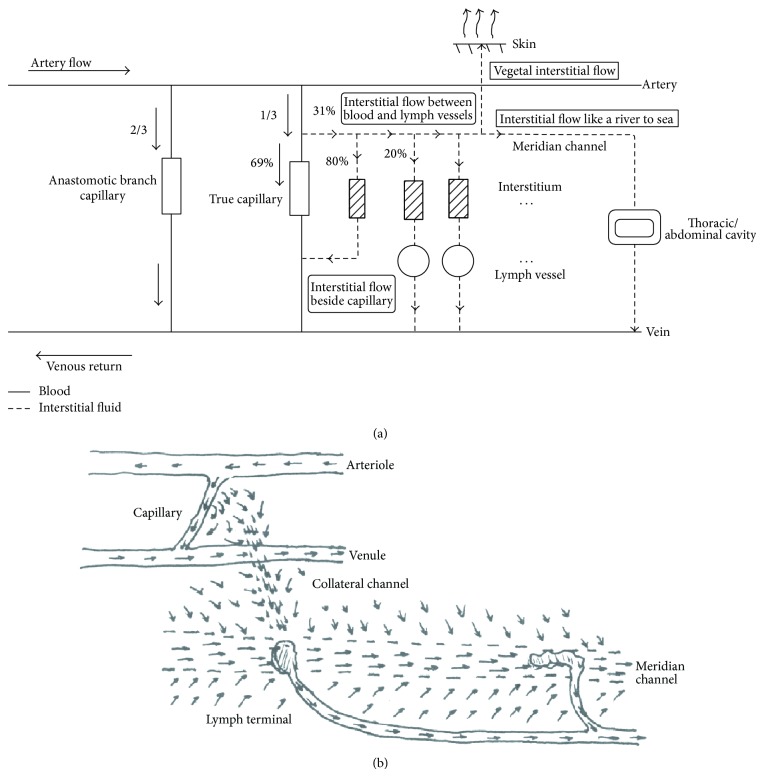
(a) General four kinds of interstitial flow on human body. (b) Interstitial flow on balancing fluid through low resistance channel between lymph terminals.

*N*_*i*_ is the flow rate of solute *i* and ∑*N*_*j*_ is the sum of flow rate of all solvents and solutes, ∇*X*_*i*_ is the concentration gradient of solute *i*, and *D*_*i*_ is the diffusion coefficient of solute *i*. According to the law, without a convection of solvents, the solute migrates back to blood vessel or lymph vessel only through the diffusion which acts when the concentration gradient (∇*X*_*i*_) of the solute becomes large while the concentration of the metabolites from a cell has become higher and destroy the milieu interne at the moment. A cell far from the vessels is more difficult to transport the metabolite without the help of solvent convection, that is, the interstitial flow. On the contrary, it is helpful to keep the milieu interne homeostasis when interstitial flow exists and higher efficiency if the interstitial flow is well organized. Four regular flows of IF could be speculated (Figure 7(a)). Interstitial flow beside capillary is the shortest route which conducts 80% of IF. There is 20% of IF flow between blood vessel and lymph vessel along the low resistance channel which cannot be totally collected by the nearest lymph terminal as the pressures of IF and lymph are similar. The IF can therefore flow through the channel which is named “river” to thoracic, abdominal, or articular cavities which are named “sea” in TCM. So the flow is simulated as the flow of a river to the sea. The relation between low resistance channel and lymph vessel is similar to a drainage ditch and drain pipes. The water coming from capillary will firstly converge on low resistance channel and move to a lymph terminal. Whenever it is not totally absorbed in the terminal or the terminal gets stasis, the IF will move to the next lymph terminal through the channel, keeping homeostasis in a large scale (Figure 7(b)). As no lymph vessel in skin, the interstitial flow will not go back into the body but will evaporate from skin surface to the air with a volume of 1-2 liters per day. The unidirectional flow is similar to the water movement in plants. So it is named vegetal interstitial flow. More complex and organized models of interstitial flow, such as a circle around 12 meridians or a circle around governor meridian (GV) and conceptual vessel meridian (CV), have been suggested in the theory of TCM which need to be proven step by step.

Apart from keeping homeostasis of milieu interne, many physiological functions have been found for interstitial flow, such as preventing edema, extracellular matrix reorganization, cell migration, capillary morphogenesis, and immunity and peripheral tolerance which have been summarized in the excellent review by Aukland and Reed in 1993 [23] and Wiig and Swartz in 2011 [64].

## 8. Summary and a Vision of Futurity

Searching for the reality of human being is an exciting and arduous experience. It is gratifying when the beautiful model of IF channel and flow appeared eventually in front of man's view. This review integrates many experimental results and provides a full view of meridian essence. People from east and west can dialogue with each other by translating Qi and Qi-channel to IF flow and IF channel and well understand why acupuncture and many related therapies exert their effects. It is very difficult to find another model which coincides with most medical experiences and the classic literatures in TCM and has been supported by many experiments.

IF and extracellular space are less studied in the west, leading a large blind area in our knowledge. The micropathway through which IF moves is called tissue channel which was firstly revealed by Casley-Smith and Vincent in 1978 [65] but only in brain tissue in which the lymph system is absent. As the techniques of observing tissue and interstitial flow are absent in peripheral tissue, the distribution of tissue channel and the pattern of interstitial flow in our body have been kept unknown until now which should be explored by both Chinese and foreign scientists.

So far, the method of measuring hydraulic resistance using a single WIN needle connected to two pressure transducers is good for animal study and has been used in many experiments. Further techniques should be developed such as imaging the flow of IF directly and showing the microstructure of meridian channel while overall logically analyzing and testing the theory of TCM on Qi and meridian is the first and foremost task. Proving the functions of different meridians is also important which could be done by blocking technique established by the authors [39]. Nevertheless, at present, it is most valuable and urgent to apply the new theory to the field of healthcare as many people are suffering from chronic diseases and unsuitable treatments. The meridian study based on the discovery of LHRC will open a new window to understand traditional Chinese medicine and to bring together Eastern and Western medicine.

## Figures and Tables

**Table 1:** The concepts in meridian system when combining meridian-collateral with entitative structures.

		Meridian	Collateral	Subcollateral	Joint
Channel system	Blood vessel	Meridian vessel	Collateral vessel	Tiny vessel	Vessel crotch
	Interstice	Meridian interstice	Collateral interstice	Striae	Muscle crotch

Supporting system	Sinew	Meridian-sinew	Collateral sinew/tubercle		Sinew Joint/muscle insertion
	Skin	Skin meridian			Skin Joint/plica
	Skeleton	Long bone	Short/flat bone		Scleromere
